# Molecular Endocrinology and Metabolism in 2021: What’s New

**DOI:** 10.3390/ijms222413375

**Published:** 2021-12-13

**Authors:** Manfredi Rizzo

**Affiliations:** School of Medicine, Department of Health Promotion, Mother and Child Care, Internal Medicine and Medical Specialties (Promise), University of Palermo, 90100 Palermo, Italy; manfredi.rizzo@unipa.it

The last two years, despite the very serious COronaVIrus Disease-2019 (COVID-19) pandemic, have been quite productive in the field of molecular endocrinology and metabolism and our journal section has contributed extensively on that. Several publications were about steroid hormones, such as estrogens and progesterone [1], and new special issues in the section “Molecular Endocrinology and Metabolism” of the International Journal of Molecular Sciences are currently accepting contributions dealing with the role of estrogen receptors in health and disease [2] and with the in vivo steroid synthesis and metabolism in healthy and pathological conditions [3]. Other authors focused on the musculoskeletal system, discussing cellular and animal models currently available for exploring skeletal muscle metabolism and endocrine function [4], and about the relationship between old age and muscle wasting in an effort to highlight the modifications in skeletal muscle metabolism associated with aging and physical activity [5].

Osteoporosis has also been a favorite topic of investigation, even with its connections to vitamin D deficiency and allergy [6,7]; in this context, a Special Issue on molecular advances in osteoporosis research still accepts valuable contributions in the section “Molecular Endocrinology and Metabolism” of the International Journal of Molecular Sciences [8]. Yet, the greatest attention has been dedicated to diabetes, insulin resistance and obesity, and their implications on cardiovascular and metabolic consequences [9,10,11]. It has also been emphasized that the role of lipid and glucose metabolism and chronic inflammation in the diabetes mellitus–atherosclerosis connection [12]. This is perfectly in line with the evidence that some atherogenic lipid subfractions, such as small dense low-density lipoproteins, are very closely linked to cardiovascular diseases [13], with a key role of some inflammatory cytokines, such as resistin [14].

This extensive interest over the last two years on the molecular aspects of diabetes, obesity, and their cardiometabolic consequences is not surprising, since a higher number of cardiovascular complications have been reported during the COVID-19 era [15]. In addition, it has been observed a bidirectional relationship between diabetes and COVID-19: diabetes is associated with an increased risk for the most severe forms of COVID-19, hospitalization in intensive care units, and related mortality; yet, at the same time, patients with COVID-19 have shown new onset of diabetes [16,17,18].

This emphasizes the importance of investigating the molecular mechanisms of diabetes and other metabolic alterations, such as obesity, that can increase cardiovascular risk; new Special Issues in the section “Molecular Endocrinology and Metabolism” of the International Journal of Molecular Sciences are currently accepting contributions dealing with the mechanisms of insulin resistance and adipose tissue dysfunction [19]; obesity, genes, and obesity-related disorders [20]; the metabolic syndrome, from molecular mechanisms to novel therapies [21]; and the advances on pathophysiology and therapies of type-2 diabetes [22]. Notably, novel antidiabetic therapies glucagon-like peptide 1 receptor agonists (GLP-1 RAs), that are effective agents with significant cardiometabolic benefit [23] beyond traditional glucose-lowering drugs [24], seem to have a molecular mechanism that may interfere with COVID-19 infection and severity [25].

In summary, the section “Molecular Endocrinology and Metabolism” of the International Journal of Molecular Sciences is committed to continue to publish high-quality articles in the field, in order to make a significant contribution to the international scientific and medical community. There are many fields that are still awaiting to be covered, such as: (a) novel insights into physiology, pathophysiology, and therapeutics; (b) cellular interactions and factors involved; (c) cross-disciplinary and integrative studies; and (d) comparative aspects of endocrinology. We can, therefore, use this difficult COVID-19 pandemic time as an opportunity to provide the best updated scientific information and valuable novel data.
